# Political economy challenges in nutrition

**DOI:** 10.1186/s12992-016-0204-6

**Published:** 2016-11-05

**Authors:** Yarlini Balarajan, Michael R. Reich

**Affiliations:** 1 Present Address: Nutrition Section, Programme Division, United Nations Children’s Fund, 3 UN Plaza, New York, NY 10017 USA; 2 Department of Global Health and Population, Harvard T H Chan School of Public Health, Boston, MA 02115 USA

**Keywords:** Nutrition, Policy reform, Political economy, Governance

## Abstract

**Background:**

Historically, implementing nutrition policy has confronted persistent obstacles, with many of these obstacles arising from political economy sources. While there has been increased global policy attention to improving nutrition in recent years, the difficulty of translating this policy momentum into results remains.

**Discussion:**

We present key political economy themes emanating from the political economy of nutrition literature. Together, these interrelated themes create a complex web of obstacles to moving nutrition policy forward. From these themes, we frame six political economy challenges facing the implementation of nutrition policy today. Building awareness of the broader political and economic issues that shape nutrition actions and adopting a more systematic approach to political economy analysis may help to mitigate these challenges.

**Conclusion:**

Improving nutrition will require managing the political economy challenges that persist in the nutrition field at global, national and subnational levels. We argue that a “mindshift” is required to build greater awareness of the broader political economy factors shaping the global nutrition landscape; and to embed systematic political economy analysis into the work of stakeholders navigating this field. This mindshift may help to improve the political feasibility of efforts to reform nutrition policy and implementation—and ensure that historical legacies do not continue to shape the future.

## Background

At this time of heightened interest in nutrition and its positioning in the post-2015 development agenda, it is worthwhile to reflect on some of the challenges that have historically faced nutrition policy reform, so that these may inform future actions. As we explain in this paper, many obstacles confronting the adoption and implementation of nutrition policies, plans and programmes arise from political economy sources. Thus there is the need to look beyond the technical aspects of nutrition and consider the broader political and economic issues—such as those relating to power, institutions, incentives, ideas, interest groups—that shape nutrition actions. Here, we describe political economy themes emanating from the literature and use them to frame the persistent challenges operating in global nutrition. These political economy challenges will need to be addressed in order to increase the political feasibility of efforts to translate momentum for nutrition into sustained results.

### Political economy themes in nutrition policy

In the field of nutrition, political economy work has been slow to develop. One early paper by Field and Levinson [1] emphasized the “dynamics of political commitment” for nutrition and development, noting that “major nutrition allocation decisions will be political decisions” ([1], 61). Many of the themes introduced by Field and Levinson in 1975 appeared in more detail—nearly two decades later—in the book by Pinstrup-Andersen [2] on the political economy of nutrition. And ten years after that, a publication on the problems of “combating malnutrition,” produced jointly by the World Bank and the United Nations Children’s Fund, examined a number of themes of political economy again [3]. More recently, however, several studies have made significant progress in applying a political economy approach to better understand the political economy themes described above affecting nutrition policy [4–9].

**Fig. 1:**
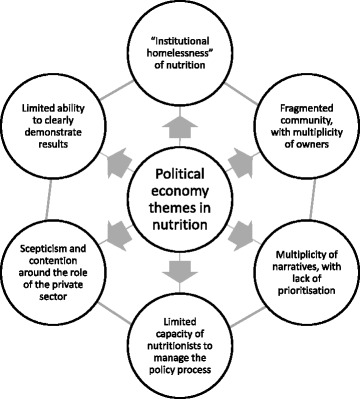
Six political economy themes in nutrition policy

Our purpose here is not to provide a detailed review of the literature on the political economy of food and nutrition security, which is provided elsewhere [7, 10], but rather to reflect on the key themes that emanate from this body of work. From our review of the literature [7], we identify six major political economy themes (Fig. 1)—these themes are highly interrelated and extend from global to sub-national level—and together create a complex web of challenges to achieving nutrition security.

First, nutrition does not have a natural institutional home; it is often “homeless” in government [2, 11]. In many cases, it does not fit cleanly into a single government agency. From country experiences, this “home” can be within supra-sectoral bodies (such as the Prime Minister’s Office or Vice President’s Office); in line ministries (such as the ministry of health or ministry of agriculture); in cross-cutting ministries or bodies (such as the ministry of development or ministry of planning); or in independent bodies. The location and characteristics of the institutional home is typically shaped by country-specific context, ideas, stakeholders, and importantly, history and politics [12]. As a result, there is often a lack of strong political leadership for nutrition, lack of strong ownership and accountability, and a lack of effective advocacy for nutrition. The fuzzy roles and responsibilities about where nutrition belongs in the government bureaucracy create obstacles to policy innovation and adoption, and to policy implementation.

At the global level as well, nutrition is claimed by several international agencies, including the Food and Agriculture Organization, International Fund for Agricultural Development, United Nations Children’s Fund, the World Food Programme and the World Health Organization, and other members of the United Nations System Standing Committee on Nutrition. Levine and Kuczynski’s [13] institutional analysis of global agencies for nutrition pointed to the lack of an effective policy community among the agencies concerned with nutrition, due to “incoherence, lack of institutional leaders, and persistent underfunding”. However, the subsequent rise of the Scaling Up Nutrition (SUN) movement and REACH (Renewed Efforts Against Child Hunger and undernutrition) initiative have sought to better align the work of these agencies in nutrition. Recent independent evaluations of these initiatives nevertheless reveal mixed reviews on improvements in UN coherence, highlighting the longstanding issues of competition, overlap and inefficiencies [14, 15]. On a more positive note, the first United Nations Global Nutrition Agenda endorsed by the agencies with a core mandate in nutrition was launched in 2015; this provides a shared vision and framework for action for the UN system [16]. Also, the findings and recommendations from the recent evaluations are shaping the new SUN strategy and UN Network for SUN strategy, to be launched in 2016.

Second, the nutrition system and nutrition community are fragmented, from global level to community level. As the final paper of the 2008 Lancet Series on Maternal and Child Undernutrition bluntly stated in its key messages, “The international nutrition system—made up of international and donor organisations, academia, civil society, and the private sector—is fragmented and dysfunctional” (Morris et al. [17], 608). Two decades earlier, Pinstrup-Anderson recognized this fragmentation within the “nutrition community,” with multiple sub-groups competing to get their view of nutrition accepted by others [2]. Levinson identified five sub-groups in the nutrition community: 1) Laboratory or Clinic-Based Nutrition Scientists, 2) Activist Nutritionists, 3) Development Economists and Planners, 4) Social Science Activists, and 5) Nutrition Practitioners [11]. This has since expanded to include public health nutritionists, agricultural specialists and other sub-groups hailing from diverse disciplinary backgrounds. These sub-groups cover different government agencies, many private sector organizations, including for-profit companies, as well as advocacy groups and households and communities.

Building coalitions across these different and disjointed groups, with their own perspectives and interests, is a time-consuming and challenging process for nutrition policy reform. The fragmentation perpetuates unclear lines of ownership and the lack of accountability for results [9]. In part, SUN was an effort to strengthen the global nutrition system and bring together and harmonise the agendas and actions of the multiple stakeholders in nutrition, under the umbrella of different networks. While it has been successful in doing this and advocating for multisectoral, multistakeholder approaches, there is still room for improvement [14].

One particularly strong disconnect exists between agriculture, nutrition, and health, with the three sectors “often moving along parallel paths that rarely seem to meet” despite efforts to “bend these paths more toward one another.” [18] It is becoming clearer that more gains can be achieved—in both nutrition and in complementary sectors—if sector-specific work also aims to address the underlying determinants of nutrition [19]. While the rationale for multisectoral actions is clear, evidence to support this is still limited, and more systematic research needed [20]. From a programmatic perspective, the adage of “planning multisectorally, implementing sectorally and reviewing multisectorally” [21] can guide coordination, reduce efficiencies and mitigate risks and/or harm. [19] Such practice can help support sectoral and geographical convergence (including targeting), clarify roles and responsibilities, and promote a stronger focus on results [22].

Third, the multiplicity of stakeholders involved in nutrition policy creates multiple narratives around nutrition approaches. For example, Rogers [23] identified eight different narratives about nutrition. There is ambiguity about what the priorities should be in the “food and nutrition security” space, and different policy communities and institutions conceptualize and interpret this space differently, as well as the relative importance of their role in this space. Players in the nutrition field can have sharply different perspectives on what the problem is and what the solution is.

These evolving narratives are captured by Jonsson’s description of seven historical periods in the development of the nutrition field, with each period promoting a single dominant paradigm of how to address malnutrition [24]. For example, over time, groups focused on inadequate consumption of food as the core problem and on food supplementation programs as the main policy solution; then groups focused on poverty as the core problem and on poverty alleviation as the main solution; then groups focused on food as a human right as the core problem and on government obligations to fulfil that right as the solution. These different narratives about malnutrition in part stem from the complex multilevel determinants of nutritional status, which become embodied in different framing efforts that cut across, bring together, or divide the multiple stakeholders involved. Moreover, the evidence to support these narratives varies in both level and quality [20, 25]. This can cause tension between different groups—for example, champions of nutrition-specific interventions cite the high-quality evidence for efficacy, effectiveness, and cost-benefits to strengthen their arguments, and point to the limited evidence available for nutrition-sensitive interventions, such as agricultural interventions, on improving nutrition outcomes—﻿thus exacerbating existing conflicts and differences in opinions.

An earlier survey of over 500 nutritionists revealed that “infighting of nutrition community and absence of consensus on priorities” was the most commonly cited disappointment among these practitioners [26]. The inability of the nutrition community to communicate issues simply and clearly to policymakers and resolve internal conflicts may have constrained collective efforts to advance nutrition policy. This is in contrast to policy communities for other global health issues, notably HIV/AIDs, which have successfully commanded the attention of global and national policy makers—despite having a less severe disease burden than malnutrition [27]. The ability of the HIV/AIDs community to strategically frame both problems and solutions and to change the narrative to respond to the evolving context [28], presents important lessons for nutrition that are yet to be realized.

Fourth, the limited capacity of nutritionists to engage in the broader political dynamics of managing nutritional policy has been highlighted. This characteristic was reported by Field and Levinson in [1], and it still persists, illustrated by a more recent complaint about the limited training that nutritionists have in managing the politics of the nutrition policy cycle [4]. Nutrition experts, at least according to some observers, are more interested in the science of nutrition than in the messy business of shaping, promoting, and implementing nutrition policy [29]. This reflects Alan Berg’s earlier diagnosis of the nutrition community’s negligence in “preparing people to work operationally in nutrition” [30]. More recently, there have been attempts to assess capacities and gaps in nutrition more systematically and to identify the skills required to respond to existing and emerging needs in nutrition [31, 32]. However, this debate has not fully addressed how such capacity in practical politics can be developed, institutionalised, scaled up or funded in the nutrition field.

Yet, a key issue remains: the relative lack of power of nutritionists in promoting policy reforms. The nutrition profession is not as organised or mature as other professional groups, such as physicians, competing in the health and development space. The relative lower professional standing of nutritionists and their discordance makes it much harder to establish credibility and command the attention of decision-makers.

Fifth, the role of the private sector in achieving nutrition security has been, and continues to be, a highly contentious issue. While there is acceptance that the private sector in its various forms does have a role to play, there is little consensus on what this should look like and how it should be regulated [33]. Many international organisations and non-governmental organisations remain deeply sceptical of the private sector. Much of this scepticism relates to a longstanding issue around compliance with ethical codes of conduct, particularly the International Code of Marketing of Breast Milk Substitutes and its associated subsequent resolutions. Lack of trust continues to stymie the nutrition community’s engagement with the private sector, and ongoing tensions with the food industry may contribute to potential partnerships with other private sector enterprises, for example in service delivery, technology and innovations, not being fostered [14].

The nutrition field is also fraught with ethical issues, and this has become increasingly complex as attention has expanded to malnutrition in all its forms, including overweight and obesity [34]. Balancing public health needs with commercial interests and managing conflicts of interest requires transparent systems and mechanisms. Steps have been taken to improve governance for public-private partnerships for nutrition, including the UNSCN Private Sector Engagement Policy [35], and more recently the SUN Business Network’s toolkit for engagement with the private sector (Guide to Business Engagement) and other guidance [36, 37], but contention remains. Compared to other networks in the SUN Movement, the SUN Business Network has been slow to start and not yet achieved what it set out to do (in part due to resource constraints), especially at country level. Of the challenges identified in the independent evaluation of SUN, “mistrust and opposition” to the private sector was perceived to be the hardest to resolve and likely to take years to address [14].

The sixth theme relates to the challenge of demonstrating and attributing results to the implementation of nutrition policy. Improving nutrition in practice is complicated and context specific; it requires careful strategic planning, informed by the situational analysis (including political and economic analysis), with prioritization of programmatic interventions to be funded with limited resources. As with other fields, there has been persistent difficulty in translating knowledge into practice and demonstrating tangible results at scale. This theme appeared in the early paper by Field and Levinson [1], and has continued to recur [8, 30]. Leroy and Menon called for more research in nutrition on moving from “efficacy to public health impact.” They argue that the lack of research on implementation is due to both the “lack of funding and limited expertise and/or interest among biologically or clinically oriented scientists” ([29], 628). With nutrition’s multicausal aetiology, it is both methodologically and practically challenging to address confounding and attribute results to specific actions.

Decision-makers and donors like to see tangible results and the hitherto lack of progress on nutrition outcomes has trapped nutrition in a “low priority cycle” [9]: “Poor [programmatic] results feed back into the low visibility for nutrition, as they are not attractive to political entrepreneurs and public officials to take them up in their own portfolio of accomplishments. This in turn creates fewer incentives for further investments in the area” [9]. More recently, however, documenting success stories in nutrition [38, 39] and learning from other public health experiences [40, 41], has enabled the sharing of lessons and demonstrated that success can be achieved at scale in relatively short time frames; although the challenge of attributing results to specific interventions remains.

## Discussion

### Political economy challenges to achieving nutrition security

The six interrelated political economy themes identified above present significant challenges to moving nutrition policy forward. These challenges can be described as the leadership challenge, resulting from nutrition’s institutional homelessness and the limited capacity and power of nutritionists to manage the policy reform process; the sectoral and coordination challenge created by the need to manage the different powers, positions and perceptions of multisectoral stakeholders in the global nutrition system; the accountability challenge, resulting from the multiplicity of owners and shared collective responsibility (or lack thereof) for results; the framing challenge, as multiple narratives are used which can muddy how the nutrition community and those they are trying to influence perceive the issue and its solutions; the hierarchy challenge, stemming from the lower standing and power of nutritionists compared to other health professionals vying for the attention of policymakers; and the demonstrating effectiveness challenge, resulting from the difficulty of demonstrating quick easily attributable wins within short political cycles.

These six political economy challenges are interrelated, with the relative importance of each one varying according to context. However, it is important to note that it may be possible to mitigate or overcome these challenges by developing tailored strategies. Table 1 summarises these challenges and potential strategies.

**Table 1:** Political economy challenges facing nutrition policy reform

Political economy challenges^a^		Examples of strategies that could be used by national nutrition institutions/governments to overcome political economy challenges in nutrition
Leadership challenge	Mainstreaming applied political economy analysis is a means to propose context-specific actions (illustrated by the examples of strategies in the next column) that can address the political economy challenges for nutrition	E.g. Actively support policy entrepreneurs or “nutrition champions” to advocate for nutrition and develop feasible policy solutions (e.g. by providing relevant data and evidence to strengthen the case for investment in nutrition or policy development).
Coordination challenge		E.g. Establish common understanding of a multisectoral approach to improve nutrition; and create appropriate incentives to align different sectoral/line ministries and stakeholders to scale up nutrition-specific and nutrition-sensitive actions.
Accountability challenge		E.g. Establish clear roles and responsibilities of different ministries and stakeholders in policy development and implementation, with mechanisms to monitor performance in a transparent way.
Framing challenge		E.g. Conduct evidence-based nutrition situation analysis and gain consensus on key nutrition problems and solutions; and establish priorities for action.
Hierarchy challenge		E.g. Invest in capacity development of national nutritionists, including as policy champions, and train in managing the policy process.
Demonstrating effectiveness challenge		E.g. Invest in national information systems that include nutrition-relevant indicators and regularly monitor and evaluate nutrition plans and their implementation.

^a^These challenges are interrelated, overlapping and context-specific

First is the leadership challenge, which can result from the lack of an authoritative institutional home for nutrition, and compounded by the limited power and capacity of nutritionists to manage the policy reform process. At the national government level, strong leadership for nutrition requires institutional commitment. This is perhaps why lessons from several countries note greater success when nutrition is led by supra-sectoral institutions, such as the office of the president or prime minister, or by line ministries when they are given authority and supported by senior government officials [22, 42]. Once institutional commitment to nutrition has been established, it provides some resistance to change, and can create a buffer from shifting priorities and short-term electoral cycles [43]. Depending on the context, certain political strategies may be effective in creating or maintaining the power of a lead institution for nutrition.

It is likely that this leadership challenge extends from national to sub-national level, depending on the type and extent of decentralization in a country [44]. However, there are few analyses of sub-national institutional leadership for nutrition, which is critical for the implementation of national nutrition policies and plans.

At the individual level, “nutrition champions” or policy advocates are critical to advocate for, and promote attention to, nutrition. Again, depending on the context, there are a number of strategies that could be used to support champions. For example, providing relevant data and evidence could support strategic advocacy and shape how nutrition and its solutions are perceived. This could be data pertaining to the nutrition situation to quantify the burden and convey the severity of the issue; or analysis of costs and benefits to present an investment case for nutrition.

Second is the coordination challenge, created by the need to bring together the many sectors and stakeholders in nutrition and manage their varying powers, positions and perceptions of nutrition. While the importance of multisectoral approaches has enjoyed a contemporary reemergence, putting this into practice remains difficult [22]. In fact, there are surprisingly few success stories of sustainable multisectoral nutrition programmes and little evidence on how to guide actions to work multisectorally, despite this challenge being recognized for decades. Recently, however, there have been several case studies conducted to address this knowledge gap, with findings and lessons learned from working multisectorally in nutrition [22, 42]. Nevertheless, more applied implementation research could help to better understand how to work multisectorally and quantify the added value of such approaches [19, 20].

In several countries, practical approaches are helping to address the coordination challenge, with concerted effort to share lessons among different countries. For example, in SUN countries, at the national level, the SUN focal point takes on a leadership and coordination role, convening stakeholders through multi-stakeholder platforms. However, there remain significant challenges to planning and implementing national plans at the sub-national level [14]. In countries where REACH is active, the initiative has facilitated multisectoral nutrition planning and catalyzed the establishment and functioning of coordinating mechanisms at national and sub-national levels, by using specific tools and approaches [15]. However, as revealed by independent evaluations of SUN and REACH, several areas for improvement remain: moreover, these approaches could be better informed by political analysis and action. For example, specific political strategies could be employed to seek common goals among different players and persuade supporters to strengthen their focus on coordinating actions to improve nutrition.

More broadly, training and sensitizing nutritionists to work multisectorally could support coalition building: in practice, this would require that nutrition champions become more fluent in the “language” of other sectors and disciplines so that they can advocate proactively and more effectively for nutrition in related policy debates and arenas, and better engage with decision-makers to influence policy reform [30].

Third is the accountability challenge, resulting from the weak governance structures and systems in nutrition, compounded by the multiplicity of owners and shared collective responsibility (or lack thereof) for results. This challenge is pervasive across the global level, as well as national and sub-national levels in nutrition [8]. At the global level, accountability of non-state actors (such as private sector corporations, civil society organizations, private foundations) is limited as institutions and mechanisms for accountability are not well established [45]. At the national and sub-national levels, without strong national and sub-national leadership for nutrition, mechanisms for accountability to citizens may not be well articulated or enforced. Furthermore, without clear multisectoral planning it may be difficult to delineate the specific actions and results for which each stakeholder is accountable for.

For nutrition particularly, managing the different interests of public and private sectors in a transparent and constructive way remains elusive. Business solutions are clearly needed to address malnutrition [8], yet historical prejudices and past experience of engagement make working with the private sector contentious and challenging. The growing epidemic of overweight and obesity presents another dimension of accountability challenges for nutrition, especially as they relate to the regulation and governance of multinational corporations in the food sector.

Recognizing this accountability challenge, some analysts have called for stronger governance in nutrition [6, 46]. However, before this can be achieved there needs to be more conceptual clarity of what governance in the nutrition sector is, what it should entail and how it can be put into place and operationalized.

Fourth is the framing challenge, resulting from the multiple narratives used that can cloud the perception of the issue and its solutions both for the nutrition community and for those they are trying to influence. Lack of consensus on priorities may have limited the credibility of nutritionists in influencing the policy agenda [26]. However, with the SUN movement, the Lancet Series and more recently, the Global Nutrition Report, greater consensus has been achieved on the key narratives and messages from the nutrition community.

Given the complexity of nutrition, multiple narratives will always exist, which may resonate in different ways with different groups outside of the nutrition community. For example, cost-benefit and cost-effectiveness analyses have been used to support the argument to invest in reducing malnutrition. Nutritionists need to learn how to strategically deploy different narratives to shape the perceptions of the nutrition problem and solutions for specific groups. For example, seeking to unify perceptions among different technical groups can bring together coalitions [*internal framing*] (for example, the United Nations Global Nutrition Agenda that outlines the joint vision for the UN agencies work in nutrition); or can promote better public acceptance of a specific definition of a nutrition issue [*external framing*] (for example, the 1,000 Days partnership [47] that has highlighted the window between pregnancy and a child’s second birthday to maximize impact of nutrition interventions delivered during this 1,000 day period).

Fifth is the hierarchy challenge, stemming from the limited power and lower standing of nutritionists, especially compared to other health professionals, notably physicians, vying for the attention of policymakers. This is perhaps more relevant to nutrition, as often the ministry of health is the line ministry housing technical expertise for nutrition and responsible for coordinating nutrition actions in health systems. Due to their professional status and expert knowledge, physicians are often seen as more authoritative and legitimate players in the policy arena. Furthermore, physicians tend to be well-mobilised and organized, such as through a national medical organization, with more ready access to decision-makers, thus constituting a powerful and influential interest group [48].

Forming strategic alliances within the nutrition community as well as with broader stakeholder groups can increase the relative power of players championing nutrition. This could involve a number of tailored strategies depending on the context that influence the relative power, position, players or perceptions of the issue [49]. For example, a power strategy could be to form a coalition of supporting players, with a recognizable name and sufficient resources, to more strongly advocate for nutrition. This was achieved in Peru with the Child Malnutrition Initiative, which brought together 14 agencies to advocate for reducing malnutrition at a critical time in the electoral cycle [42]. In another situation, a perception strategy could be used to mobilise the media or civil society groups to draw attention to malnutrition as an issue needing attention; this has been done in India, with powerful external framing of child nutrition as a problem identified by the media and supported by credible indicators of severity of the issue, and by the ‘Right to Food’ movement that has framed undernutrition in terms of constitutional rights and entitlements [50]. Supporting such social mobilization efforts to bring public attention to nutrition issues can increase the perception of the nutrition problem as important to address. In turn, this may strengthen the legitimacy of the nutrition community and boost their power to drive policy reforms.

In the long-term and more broadly, addressing the hierarchy challenge will require strategies to build capacity within the nutrition sector and ensure that adequate levels and quality of human resources for nutrition are developed to support nutrition actions in the post-2015 era [31]. This demands building a strategic and appropriate mix of skills in the nutrition community—and importantly, leveraging external expertise or partnering with others to build a team that together has a complete set of skills and expertise to push the nutrition agenda forward.

Sixth is the demonstrating effectiveness challenge, resulting from the difficulty of demonstrating quick wins in nutrition improvements within short political cycles. The complexity of tackling nutrition problems through multiple interventions makes achieving results and, importantly, measuring (or claiming) attribution to outcomes, very challenging. Even though rapid reductions in stunting and other forms of malnutrition have been achieved within the relatively short time period of a few years, the time required to see and scientifically document nutrition improvements can far exceed the short term horizons of politicians seeking re-election.

Greater emphasis needs to be placed on supporting rigorous monitoring and evaluation and investing in routine information systems; documenting experiences and lessons learned, even if in smaller geographical areas or in the context of specific projects; and investing in more strategic communication around results (for example, stories of change which convey the challenges and bottlenecks, how they were overcome and how they led to positive changes). Demonstrating clear progress and developing compelling narratives, could help dismiss the perception of the nutrition problem as being too risky or too hard to tackle, and make championing nutrition a more attractive choice for politicians.

## Conclusion

With these six entrenched political economy challenges in nutrition, being more strategic and innovative in navigating the nutrition policy landscape could help in moving the nutrition agenda forward. We argue that a “mindshift” is needed in the nutrition community to reframe the lens to think beyond technical issues. This is particularly relevant to nutritionists, who typically work in the very complex multistakeholder area of global development. This mindshift to grasp the six political economy challenges in nutrition needs to be accompanied by steps to build awareness, capacity and skills to navigate the complex nutrition policy process—from agenda setting, to policy development, policy adoption and implementation.

Generating greater awareness of political economy in nutrition and mainstreaming political economy analysis will help to devise local strategies to enhance the political feasibility of reform. Applied political economy analysis for nutrition comprises a spectrum of approaches that can be used to inform decision-making and identify political strategies for reform [7, 10]. Such political strategies broadly relate to shaping power, position, players and perceptions, and can be tailored and iterated according to the changing political context—at national and sub-national levels.

Improving nutrition globally requires managing the persistent political economy challenges affecting nutrition policy and its implementation. As we have argued previously, there is an urgent need to bring political economy analysis into the consciousness of those working in nutrition [51]. A more systematic approach to resolving the political economy challenges for nutrition will ensure that historical legacies do not continue to shape the future.

## Summary

This paper presents the political economy themes emanating from the political economy of nutrition literature and uses these themes to frame six challenges still facing nutrition reform today. These challenges relate to leadership, coordination, accountability, issue framing, hierarchy and demonstrating effectiveness of nutrition actions. We argue that a mindshift is required to build greater awareness of the broader political economy factors shaping the global nutrition landscape; and to embed systematic political economy analysis into the work of stakeholders in the nutrition field.

## Abbreviations

SUN: Scaling Up Nutrition
REACH: Renewed Efforts Against Child Hunger and undernutrition
UNSCN: United Nations Standing Committee on Nutrition
